# Risodontrypy; Or, Treatment of Exposed Nerves

**Published:** 1853-07

**Authors:** C. O. Cone

**Affiliations:** Baltimore


### Selected Articles.

### *Risodontrypy*; *Or, Treatment of Exposed Nerves.*—By C. O. Cone, M. D., of Baltimore.

[Continued from page 167.]

Case 8th. *Nov.* 4, 1851.—To-day, Mr. S., aged 27, of a sanguinous nervous temperament, consulted me in relation to a large cavity on the posterior aproximal surface of the left, second, superior bicuspid. The tooth had several times given him uneasiness. The diseased dentine readily yielded on pressure, compressing the nerve and affording great pain. I excavated the diseased dentine, exposing the nerve which was very irritable and painful. Its vessels bled freely. I proceeded to perform Hullihen's operation. The drill that I endeavored to employ in the operation was not sufficiently tempered, and bent under the effort. The refractory condition of the patient, growing out of apprehension and natural excitability, admonished me to avoid any delay in performing the operation. I therefore completed the puncture through the fang with a drill, some larger than would have been chosen for the purpose, and with a neck almost equal in size to the point of the cutting edge. Pain and irritation of the nerve ceased on the completion of the operation. The pain attending the operation was not great.

*Nov.* 11.—Saw Mr. S, every day from the time the operation was performed. But little inflammation occurred in the gum. No unusual symptom occurred to direct the attention of the patient to the tooth, further than it "felt a little sore."

*Nov.* 19.—The tooth has become very sensitive and painful to the presence of warm fluids received into the mouth.

*Nov.* 21.—Mr. S's. tooth is still painful to the presence of warm fluids, and feels a little elongated.

---

*Last summer, when on a visit up the river, we had the pleasure of seeing the above described, Blow pipe of Dr. William's. It struck us then, as a very convenient apparatus, and as we had never seen one of the kind, we requested a description for the Register. The jet is continued and plenty strong for any use in a dental laboratory.—[Ed. Reg.

*Nov.* 24.—To-day on examination, I found a small collection of matter, at the point pierced by the drill over the fang of the tooth. All pain subsided on the appearance of the pus.

*Dec.* 1.—Examined the tooth of Mr. S. No change.

*May* 9, 1852.—examined the tooth again. The discharge of pus at the mouth of the puncture is much less. Some little uneasiness in applying pressure in some directions to the crown of the tooth. The vitality of the crown destroyed.

CASE 9th. *Dec.* 6, 1851.—While excavating a cavity on the posterior aproximal surface, of the right, inferior, first molar, for Miss D., aged about 30, of a nervous sanguinous temperament, with general good health, I exposed the nerve at a small point. The exposure was attended with a good deal of pain, of an acute nervous character, which was felt on exhausting the atmosphere from the cavity. I proceeded to perform Hullihen's operation. The puncture could not be made back of the alveolus, as it would carry the drill too far down on the tooth to allow the instrument to enter the nerve chamber. The entrance of the drill into the nerve cavity, was distinctly felt with the hand, and observed by an exclamation of pain by the patient. As I withdrew the drill, I felt it break. An examination of the puncture presented the broken end of the drill, exposed about half a line beneath the dentine of the tooth. Could not dislodge it. All pain of the operation quickly subsided. The atmosphere could now be withdrawn from the cavity without pain. Yet there was sensibility of the exposed nerve, I determined under the circumstances, to perform the operation of filling the tooth which was completed at this sitting.

*Dec.* 12.—Saw Miss D. She has had no pain in the tooth operated on more than in other teeth, when the cavity is sensitive, in the operation of plugging. The gum is slightly swollen about the puncture. Pressure on the crown of the tooth in all directions induces no pain whatever. Tooth sensitive, when ice-water is brought in contact with the plug. Patient expressed more confidence in the permanent success of the operation than I feel.

*May* 17, 1852.—To-day I examined Miss D's. tooth on which I operated breaking the instrument. The tooth has given her no inconvenience. It is still endowed with vitality.

The puncture through the gum has not closed. No inflammation in the gum. On exploring the puncture, could detect where the drill had penetrated the tooth, after which the bristle met with resistance. There is no discharge from the puncture.

*July* 9, 1852.—Had an opportunity of making inquiry from a member of the family, and Miss D's. tooth still remains comfortable, without offering any kind of offense.

Case 10. *Dec.* 13, 1852.—I exposed a nerve in a cavity on the anterior approximal surface of the left, superior, central incisor, for Mrs. J., aged about 28, of a nervous bilious temperament, and precarious health. In addition to her usual feeble health, the patient has recently recovered from an attack of typhoid fever. The tooth in which I exposed the nerve has given uneasiness, but no absolute tooth ache, I performed on this Hullihen's operation. It was not attended with much pain. The exhaustion of the atmosphere from the cavity, after the operation, was not attended with the same degree of pain as before it was performed. On examining my drill, I found the extreme point broken. I doubt not that it is somewhere in the artificial canal, but cannot discover it by examination.

*Dec.* 17.—To-day I examined the tooth of Mrs. J. The gum is but little inflamed. To pain has been felt in it, and pressure can be applied to the crown in all directions without pain. To-day I exposed a nerve in a cavity in the anterior approximal surface of her right, superior central incisor. The tooth had given the patient some uneasiness, but not tooth-ache. I performed Hulihen's operation which was attended with but little pain. Could exhaust the atmosphere from the cavity after the operation without as much pain as before it.

*Jan.* 13, 1852.—I was called on by Mrs. S. Complains of pain in the left, superior latteral incisor, which I had plugged on the 23d of December last. The plug was introduced in the anterior approximal surface. No symptom attended the preparation of the cavity or during the operation of plugging to lead to the suspicion that the nerve of the tooth was exposed. The tooth began to pain her on the 11th, and gradually increased until now; pulsating pain is felt in the crown of the tooth. No elongation is felt; nor is pressure on the crown of the tooth in any direction attended with pain. The gum about the tooth is reddened, and in places excoriated by some stimulating application made by the patient. I performed Hullihen's operation. The drill was shorlty followed by a drop or more of slightly colored serum. The pain immediately abated. The two central incisors on which I had operated had given her no pain. The puncture over her right incisor had almost closed, and the point could only be detected by a difference of color. The puncture over the left central incisor has not closed and its location can be distinctly recognized.

*Jan.* 19.—Mrs. J. informed me that the left, superior, lateral incisor, gave her some pain yesterday. The gum about the puncture is slightly swollen. She tells me that cold or warm drink taken into the mouth produces pain in the three incisors operated on. No pains experienced by applying pressure to the crowns of either of these three teeth.

*Jan.* 20.—I exposed the nerve in a small fissure when excavating a cavity on the masticating surface of the superior, right molar, in the mouth of Mrs. J. I performed Hullihen's operation. The drill entered the tooth between the neck and the alveolus, just where the bucal fang united with the crown of the tooth. The operation was attended with considerable pain after which none was felt on exhausting the atmosphere from the cavity.

*Jan.* 22.—Mrs. J. informs me that no discomfort followed the operation on the molar. The tooth and gum on examination present no symptoms of inflammation.

*Feb.* 15.—Mrs. J. complains of pain in the molar which had its nerve exposed. No inflammation in the gum; no pain produced by pressure in any direction when applied to the crown of the tooth. The pain did not continue long at any time but occurred at intervals, but was not of a throbbing character.

*March* 29.—To-day Trs. J. called at my office with her face swollen. She had suffered pain for two days, in the right superior central incisor. This tooth was sensitive to the least pressure. There was a collection of pus in the tissue about the gum—gave it vent by lancing, which afforded, immediate relief.

*May* 17.—To-day I examined the teeth in Mrs. J's mouth on which I performed Hullihen's operation. The nerves in the right superior central incisors, and the left, superior lateral, were separated. The teeth have changed their color, and puss has been forced from the puncture of each, which has not closed in the least. The right, superior, second molar still retains its color. The puncture in the gum has almost closed.

CASE 11th. *Jan.* 15, 1852.—Miss DeB., aged about 19, is of nervous bilious temperament, and general good health, consulted me in relation to refilling the posterior approximal surface of the right, superior central incisor. In excavating the cavity I exposed the nerve in a fissure of the chamber. The whole operation of excavating the cavity was attended with a good deal of pain. The tooth had never before given the patient any discomfort. The atmosphere could not be exhausted from the cavity without a severe nervous pain being felt through the whole tooth. Performed Hullihen's operation. This was done with an instrument larger than the usual size of the nerve canal at the point perforated, and at the age of the patient. The operation was marked by considerable pain. Proceeded to fill the tooth.

*Jan.* 22.—To-day I had an opportunity of examining the tooth of Miss DeB. She had experienced no discomfort except when very cold liquids were received into the mouth, when the tooth would become painful for a few minutes. No pain on pressure being applied to the crown of the tooth. There was considerable inflammation immediately about the puncture in the gum.

*March* 4.—To-day I examined the tooth operated on in the mouth of Miss DeB! The puncture is closed and can only be detected by a slight difference in the color of the tissue, and without a knowledge of the operation having been performed, would not be detected on careful examination. The tooth has given no discomfort, and feels healthy to the patient.

